# Crosslinking Mechanism on a Novel *Bacillus cereus* Transglutaminase-Mediated Conjugation of Food Proteins

**DOI:** 10.3390/foods11223722

**Published:** 2022-11-19

**Authors:** Hongbin Wang, Yuanfu Zhang, Zhaoting Yuan, Xiaotong Zou, Yuan Ji, Jiayi Hou, Jinfang Zhang, Fuping Lu, Yihan Liu

**Affiliations:** 1 Key Laboratory of Industrial Fermentation Microbiology, Ministry of Education, The College of Biotechnology, Tianjin University of Science and Technology, Tianjin 300457, China; 2 Tianjin Key Laboratory of Industrial Microbiology, Tianjin 300457, China

**Keywords:** transglutaminase, enzymatic characterization, structural analysis, crosslinking properties, protein substrate crosslinking mechanism

## Abstract

Until now, *Streptoverticillium mobaraense* transglutaminase (TG) is the only commercialized TG, but limited information is known about its selection tendency on crosslinking sites at the protein level, restricting its application in the food industry. Here, four recombinant *Bacillus* TGs were stable in a broad range of pH (5.0–9.0) and temperatures (<50 °C), exhibiting their maximum activity at 50–60 °C and pH 6.0–7.0. Among them, TG of *B. cereus* (BCETG) demonstrated the maximal specific activity of 177 U/mg. A structural analysis indicated that the Ala147-Ala156 region in the substrate tunnel of BCETG played a vital role in catalytic activity. Furthermore, bovine serum albumin, as well as nearly all protein ingredients in soy protein isolate and whey protein, could be cross-linked by BCETG, and the internal crosslinking paths of three protein substrates were elucidated. This study demonstrated *Bacillus* TGs are a candidate for protein crosslinking and provided their crosslinking mechanism at the protein level for applications in food processing.

## 1. Introduction

Proteins perform a vital role in food quality as a critical fraction of food products. Nowadays, protein modification technologies attract more and more attention due to their importance in satisfying the consumers’ various food requirements [1]. Compared to chemical modification, protein modification using enzymatic techniques demonstrates lots of advantages, such as a low frequency of side reactions, high reaction specificity, and the lack of a need for chemical solvents (environmental friendliness) or high-pressure and high-temperature conditions [2]. In this context, protein crosslinking, which is described as “the process of joining protein molecules by inter or intra-molecular covalent bonds”, plays an important role in determining the functional characteristics of foods [3].

Transglutaminase (EC 2.3.2.13, TG) leads to inter- and intra-molecular crosslinking through catalyzing the generation of ε-(g-glutamyl) lysine ‘‘isopeptide” covalent bonds in proteins. Currently, TGs have been discovered and characterized in prokaryotes and eukaryotes. Comparing with the eukaryotic counterparts, bacterial TGs demonstrate lots of obvious advantages, such as cofactor independency, small size, high stability and improved performance [4], and they have been chiefly discovered in *Bacillus* and *Streptoverticillium* strains. TG derived from *Streptoverticillium mobaraense* (MTG) is the sole transglutaminase commercialized for food processing [5]. It is mainly used for changing proteins’ elasticity and solubility, producing textured products, improving protein encapsulation capacity, emulsifying properties, and water-holding capacity, improving the nutritious value of food products by the incorporation of essential amino acids, forming heat and water-resistant films, avoiding gelation by thermal treatment, and, recently, to reduce allergenicity [6]. MTG demonstrates its stability below 40 °C from pH 5.0 to 8.0 and its maximum activity at 50 °C between pH 6.0 and 7.0 [7]. However, TG from *B. subtilis* (BSUTG) exhibits its maximum activity at pH 8.0 and 60 °C, which are more stable under wide ranges of pH (pH 5.0–9.0) and temperature (30–60 °C). Meanwhile, BSUTG and MTG have only ∼10% homology in amino acid sequences, and they also show lots of differing preference in substrates such as soy protein isolate (SPI) and whey protein (WP). Obviously, it would be helpful to meet the large number of needs of various food substrates and processing due to the diversity of TGs in nature. Hence, it would offer a novel challenge for utilizing TGs in the rising food field by exploring novel TGs. In addition, our previous study investigated BSUTG’s crosslinking characteristics on peptide levels [8]. Nevertheless, selecting food protein crosslinking sites might be affected by both the neighboring amino acids and the protein spatial structure of BSUTG. So far, limited information on the favorite amino acid crosslinking sites is known on the food protein level.

In this study, we characterized TGs derived from *B. amyloliquefaciens*, *B. cereus*, *B. safensis*, and *B. aryabhattai*. Then, molecular dynamics simulation was performed to analyze the *B. cereus* TG’s tertiary structure to explore the molecular mechanism of its catalytic activity under different temperatures. Finally, we estimated the abilities of *B. cereus* TG towards various substrates and the preferred crosslinked amino acid sites. This study facilitates the applications of *Bacillus* TGs in the food industry and the depth our understanding of them.

## 2. Materials and Methods

### 2.1. Strains, Plasmids, Materials, and Growth Media 

*B. amyloliquefaciens* TCCC 111018, *B. cereus* TCCC 111006, *B. safensis* TCCC 111022, and *B. aryabhattai* TCCC 11368 were kept in our laboratory, and they were incubated at 37 °C in liquid LB medium. *Escherichia coli* BL21 (DE3) was utilized for expressing the recombinant enzyme, and kanamycin was added when necessary. The pET-28a(+) was employed as an expression vector. 

Reagents were provided by the following sources: *N*,*N*-dimethylcasein and dansyl cadaverine [N-(5-aminopentyl)-5-dimethylamino-1-naphthalenesulfonamide, MDC] were ordered from Sigma-Aldrich Co. (St. Louis, MO, USA) and Aladdin Chemical Co., Ltd. (Shanghai, China), respectively. Bovine serum albumin (BSA, ≥98%), soy protein isolate (SPI, ≥88%), and whey protein (WP, ≥80%) were provided by Solarbio Science & Technology Co., Ltd. (Beijing, China), Macklin Biochemical Co., Ltd. (Shanghai, China), and Sigma-Aldrich (St Louis, MO, USA), respectively.

### 2.2. Strains Construction

The TG genes encoding *bamtg*, *bcetg*, *bsatg*, and *bartg* were obtained using the forward and reverse primers presented in Table S1 and genomic DNA from *B. amyloliquefaciens* TCCC 111018, *B. cereus* TCCC 111006, *B. safensis* TCCC 111022, and *B. aryabhattai* TCCC 11368 as templates. After digestion with *Nco*I and *Hin*dIII, the PCR products *bamtg*, *bsatg*, and *bartg* were inserted into the *Nco*I and *Hin*dIII-linearized pET-28a (+). However, the PCR product *bcetg* was ligated to the *Nco*I–*Not*I-linearized pET28a(+) due to the *Hin*dIII restriction site in the *bcetg* gene. The sequences of *bamtg*, *bcetg*, *bsatg*, and *bartg* were deposited in GenBank, respectively (Accession No: MN537144, MW281568, MW292483, and MW916358). *E. coli* BL21 (DE3) cells harboring the recombinant vectors pET-*bamtg*, pET-*bcetg*, pET-*bsatg*, and pET-*bartg* were utilized for protein expression. Their protein sequences were analyzed using DNAMAN for multiple protein sequence alignment, and the signal peptides were predicted by the Signal P 3.0 server (http://www.cbs.dtu.dk/services/SignaIP-3.0 (accessed on 8 June 2019)).

### 2.3. Production and Purification of TGs

Each single colony of BL21/pET-*bamtg*, BL21/pET-*bcetg*, BL21/pET-*bsatg*, and BL21/pET-*bartg* was grown at 37 °C for 12 h in 5mL of LB medium containing 50 μg/mL kanamycin with shaking at 200 rpm. Subsequently, the seed culture (1 mL) was inoculated into 50 mL of fresh LB medium with kanamycin (50 μg/mL). When OD600 of the cells reached 0.6–0.8, 0.5 mM isopropyl-β-D-1-thiogalactopyranoside (IPTG) was utilized to induce the production of recombinant TGs (rTGs) at 16 °C for 20 h. 

Cells were centrifugally collected at 8000× *g* and 4 °C for 15 m, followed by resuspension in the lysis buffer (20 mmol/L CAPS, 0.5 mol/L NaCl, 10% glycerol, 10 mmol/L imidazole, pH 8.0). Then, the cells were sonicated at 260 W with 2 s strokes and 4 s intervals. After centrifugation at 12,000× *g* for 30 min, the resulting supernatant was loaded onto a nickel–nitrilotriacetic acid (Ni-NTA) agarose column (Qiagen, Germany) which was preequilibrated with lysis buffer. After removing non-target proteins with washing buffer (20 mmol/L CAPS, 0.5 mol/L NaCl, 10% glycerol, 300 mmol/L imidazole, pH 8.0), the purified rTGs were collected after eluting with elution buffer (20 mmol/L CAPS, 0.5 mol/L NaCl, 10% glycerol, 60 mmol/L imidazole, pH 8.0). Sodium dodecyl sulfate–polyacrylamide gel electrophoresis (SDS-PAGE) was performed to analyze the purified protein, and the Bradford method was utilized toevaluate the protein concentration, with bovine serum albumin as the standard.

### 2.4. TGs Activity Assays

TG activities were measured based on the protocol described by Liu et al. [9] with some modifications. After mixing 200 μL of phosphate buffer (50 mM, pH 6.0 or 7.0, optimal pH for various TGs) and 100 μL of enzyme-incorporating MDC (12.5 μM) and *N*,*N*-dimethylcasein (0.2%), the enzymatic reaction was performed for 20 min at 50 °C or 60 °C (the optimum temperature for different TGs). The fluorescent intensity was recorded by the Fluorescence Spectrophotometer-Infinite 200PRO (Tecan, Austria) based on a previous study [8].

### 2.5. Characterization of TGs

The effect of pH on the activities of TGs was determined over the pH ranging from 5.0 to 9.0 under their optimum temperatures. The following buffer systems were applied in this work: citrate-phosphate buffer (50 mM, pH 5.0), phosphate buffer (50 mM, pH 6.0–8.0), and Tris-HCl buffer (50 mM, pH 9.0). After incubating the enzyme in the above-mentioned buffers at 4 °C for different times, the influence of pH on the stability of TGs was tested at pH = 5.0–9.0 by calculating the retained enzyme activity under standard conditions.

The optimum temperatures of TGs were ascertained by measuring the activities in the buffer of their optimal pH at differing temperatures of 30–80 °C. Thermostability was detected by keeping TGs in phosphate buffer (50 mM, pH 7.0) for various periods at 30–80 °C, and then calculating the residual activity.

### 2.6. Molecular Dynamics Simulations

The docking of TG (BCETG) from *B. cereus* TCCC 111006 with the substrate was conducted using AutoDock4.2.6 software [10]. After inputting the protein sequence of BCETG, the searching results were obtained on SWISS-MODEL (http://www.swissmodel.expasy.org (accessed on 7 January 2020)). We selected the 3D structure of TG from *B. subtilis* (BSUTG, PDB accession No. 4P8I) as the template for building the BCETG structure. In addition, we obtained the 3D structure of the MDC substrate from the PubChem Web site (http://pubchem.ncbi.nlm.nih. gov (accessed on 7 January 2020)). By observing the location of the BCETG active site (Cys117, Glu116, and Glu189), the volume of 60 Å × 60 Å × 60 Å was chosen for the center of the grid box to fit the ligand easily and cover the whole pocket of the active center. Then, MD simulation was carried out using the best conformation exhibiting the minimum binding energy for protein–substrate docking.

MD simulation was conducted with GROMACS 5.1.4 software in combination with the GROMOS96 54a7 force field parameters [11], using almost the same simulation parameters as those reported in detail [12,13]. The minimum distance of BCETG and the cube box edge was 15 Å under the periodic boundary conditions, and then water molecules filled in the box. The SPC/E model was utilized to describe water. The system was charged zero to replace the equivalent number of ions by using random water molecules. The simulation systems were optimized using the steepest decent method of 50,000 steps. Both short-range van der Waals (vdW) and electrostatic interaction (ELE) were easily truncated at 10 Å. Different temperature gradients (323 K and 353 K) were repeated in the isochoricisothermal (NVT) ensemble three times. The size of the mesh was 0.16 Å with the intercept at 12 Å. The external temperature and pressure baths were to couple MDC, protein and water molecules. In the end, each simulation was carried out for 100 ns while recording the coordinates every 2 ps. 

The trajectory of the MD simulation was evaluated with the supplementary program in the GROMACS 5.1.4 software package (SciLifeLab, Stockholm, Sweden). The typical snapshots of the BCETG tertiary structures were obtained using the Visual Molecular Dynamics (VMD) 1.9.4 software [14].

### 2.7. Protein Substrates Crosslinked by BCETG 

The BSA (1 mg/mL), SPI (8 mg/mL), and WP (1 mg/mL) solutions were made in sodium phosphate buffer (50 mM, pH 6.0). Then, the crosslinking was performed at 50 °C by mixing BCETG with protein in the concentrations of substrate (0.1, 0.03, 0.1 mg/mg) containing DTT (2 mM) for various periods (1–6 h). Finally, SDS-PAGE was utilized to characterize BCETG that catalyzed the crosslinking conditions of BSA, SPI, and WP for differing periods, as noted by Liu et al. [5,7,15].

### 2.8. Analysis of Protein Substrate’s Crosslinked Amino Acid Sites by BCETG 

The crosslinked sites in the crosslinked protein samples by BCETG were identified by liquid chromatography linked to the tandem mass spectrometry (LC-MS/MS) through analyzing the crosslinked peptides. After being boiled for 10 min to stop crosslinking, the protein samples that were crosslinked for 1 h were then denatured, reduced and digested with trypsin based on the previous method [8]. LC-MS/MS analysis was performed by a 1260 series HPLC coupled to an ESI-Q/TOF mass spectrometry (Agilent Technologies, Palo Alto, CA, USA) using the previously reported conditions [8]. The crosslinked peptides were identified and quantified using the software PLink 2 (The Institute of Computing Technology of the Chinese Academy of Sciences, Beijing, China) and the Masshunter B.08.00 of Agilent (Palo Alto, CA, USA), respectively, by processing the mgf data. The conditions used for the identification by the software PLink were as the following. Protein database: BSA for BSA protein, soybean proteins for SPI sample, and whey proteins for WP sample; protease: trypsin; variable modifications: methionine oxidation; fixed modification: cysteine carbamidomethylation; missed cleavages: no more than two; fragment ion tolerance: 50 ppm; precursor mass tolerance: 50 ppm.

## 3. Results and Discussion

### 3.1. Molecular Cloning and Genes Sequence of bamtg, bcetg, bsatg, and bartg Genes

The *bamtg*, *bcetg*, *bsatg*, and *bartg* genes showed an open reading frame of 735, 828, 738, and 813 bp, coding for a full-length protein of 244, 275, 245, and 270 amino acids with a theoretical molecular mass of 28.3, 31.4, 28.4, and 31.3 kDa (BAMTG, BCETG, BSATG, and BARTG), respectively. No signal peptide was found to locate at the N-terminus of the BAMTG, BCETG, BSATG, and BARTG after analysis using the Signal P program. The gene sequences were stored in the NCBI database, and the accession numbers of the *bamtg*, *bcetg*, *bsatg*, and *bartg* genes were MN537144, MW281568, MW292483, and MW916358, respectively. BAMTG, BCETG, BSATG, and BARTG showed 71.4%, 34.7%, 51.8%, and 38.3% of protein sequence identities with the reported BSUTG, respectively, after multiple amino acid sequence alignments (Figure S1). Meanwhile, they only shared ~10% identity with MTG. It suggested that they were probably the novel members of TG. As the first TG isolated from *Bacillus* species, BSUTG is produced to crosslink the highly resilient spore surface during sporulation [16]. In addition, it is the smallest TG produced as an active single-domain protein. Furthermore, it functions via a peculiar partly redundant catalytic dyad containing Glu115 and Cys116 or Glu187. Comparative analysis of the primary sequences indicated that BAMTG, BCETG, BSATG, and BARTG contained the conserved amino acids correspondent to Glu115, Cys116, and Glu187 of BSUTG, which were considered to play a catalytic role. However, their amino acid sequences showed a significant homology with BSUTG, and especially MTG, which could cause differing characteristics and substrate preference from MTG and BSUTG. 

### 3.2. Heterologous Production and Purification of TGs

The genes of *bamtg*, *bcetg*, *bsatg*, and *bartg* from *B. amyloliquefaciens* TCCC 111018, *B. cereus* TCCC 111006, *B. safensis* TCCC 111022, and *B. aryabhattai* TCCC 11368 in *E. coli* with a C-terminal 6 × His tag. The clear bands corresponding to the purified BAMTG, BCETG, BSATG, and BARTG were observed on the SDS-PAGE gel (Figure S2) 

### 3.3. Biochemical Characterization of BAMTG, BCETG, BSATG, and BARTG

**Figure 1:**
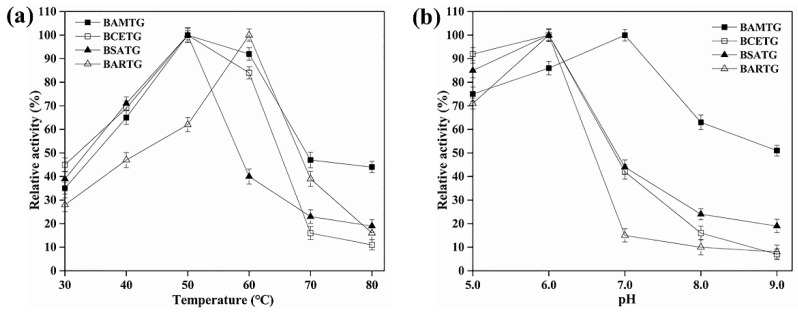
The optimum temperature and pH of recombinant TGs. (**a**) The influence of temperature on the BAMTG, BCETG, BSATG, and BARTG activities; (**b**) The influence of pH (5.0–9.0) on the activities of BAMTG, BCETG, BSATG, and BARTG. Three independent experiments were performed to calculate the values described by means ± SD.

**Figure 2:**
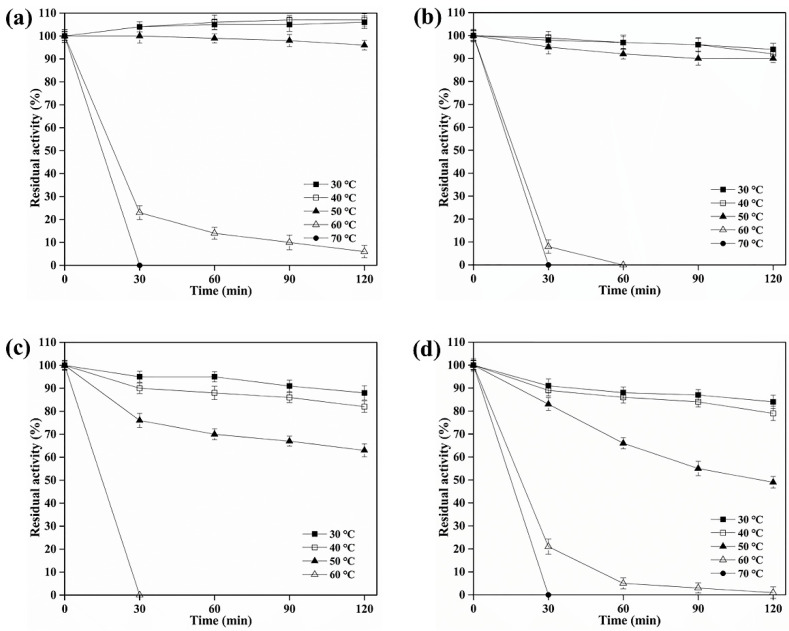
The thermostability of recombinant TGs. The effect of temperature on the stability of (**a**) BAMTG, (**b**) BCETG, (**c**) BSATG, and (**d**) BARTG. Three independent experiments were performed to calculate the values described by means ± SD.

The profile of BARTG activity as a function of temperature variation indicated its optimum condition around 60 °C (Table 1), and BARTG displayed > 40% of its highest activity between 40 °C and 70 °C (Figure 1a). MTG only showed about 60% of its maximal activity at 60 °C and showed no activity at 70 °C, though it was most active at 55 °C [9]. Additionally, BAMTG, BCETG, and BSATG showed their maximal activity at 50 °C (Table 1, Figure 1a). MTG retained merely ∼20% of its initial activity after incubating at 50 °C for 100 min [9], but BAMTG, BCETG, BSATG, and BARTG retained about 100%, 100%, 60%, and 50% of their original activities after 120 min incubation at 50 °C, respectively (Figure 2a–d). These results verify that four TGs had better thermostabilities than MTG. 

**Figure 3:**
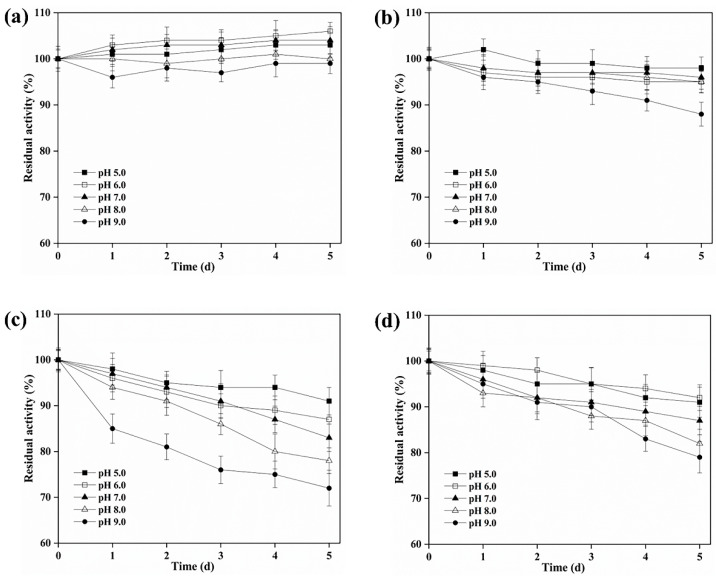
The pH stability of recombinant TGs. The effect of pH on the stability of (**a**) BAMTG, (**b**) BCETG, (**c**) BSATG, and (**d**) BARTG. Three independent experiments were performed to calculate the values described by means ± SD.

BAMTG showed over 50% activity from pH 5.0 to 9.0, and its optimal pH was 7.0, but the optimal pH of BARTG, BCETG, and BSATG was 6.0 (Table 1, Figure 1b). By contrast, MTG showed no activity at pH 9.0 [9]. Moreover, BAMTG, BARTG, BCETG, and BSATG kept stable under an extensive pH range of 5.0 to 9.0, retaining approximately 100%, > 90%, > 80%, > 70% of the initial activity after incubation for 5 d between pH 5.0 and 9.0, respectively (Figure 3a–d), which was superior to MTG with a pH stability range of 5.0–8.0 [9]. These data indicate that these TGs could be a potential candidate for various biotechnological processes under acidic–neutral–alkaline conditions. 

As the most common enzyme applied for protein crosslinking, MTG is the only commercially available enzyme utilized in the food industry. However, the biological approach might be limited by the enzymatic properties. Hence, it could be essential to provide an extensive range of available TGs for crosslinking to the food processors. These four TGs demonstrated various temperature and pH ranges for their activities and stabilities, and these properties were clearly unlike that of MTG. Thus, they were beneficial for covering the disadvantages of MTG’s properties in different types of food processing.

### 3.4. Molecular Dynamics Simulation

**Figure 4:**
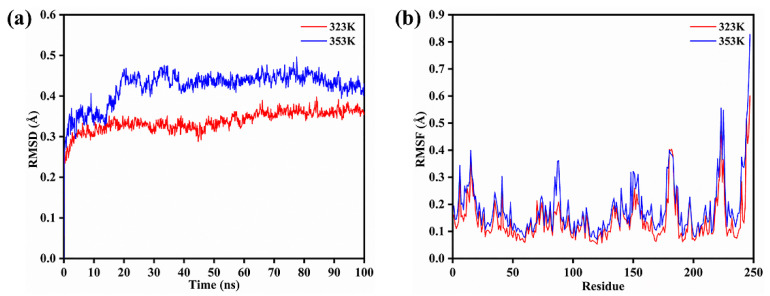
The structural changes of BCETG at 323 K and 353 K. (**a**) The root-mean-square deviation (RMSD) of BCETG at 323 K and 353 K. (**b**) The root-mean-square fluctuation (RMSF) value for each residue in BCETG at 323 K and 353 K.

According to the optimum pH and temperature, the specific activities of BAMTG, BCETG, BSATG, and BARTG were determined to be 4.3, 176.8, 2.5, and 9.1 U/mg, respectively. BCETG exhibited the highest specific activity; however, its optimum temperature (50 °C) was unremarkable. Therefore, in order to investigate the reason for the low catalytic activity of BCETG at high temperatures, MD simulation was used to analyze the structural changes of BCETG at 323 K (50 °C) and 353 K (80 °C). Figure 4a shows the change in BCETG’s root-mean-square deviation (RMSD) values at 50 °C and 80 °C as the function of simulation time (100 ns). It demonstrates that BCETG at 50 °C and 80 °C showed stabilized RMSD values after 80 ns, and BCETG showed a ~0.05 Å lower RMSD value at 50 °C than that at 80 °C, finally. This indicates that the structure of BCETG at 50 °C was more stable than that at 80 °C.

**Figure 5:**
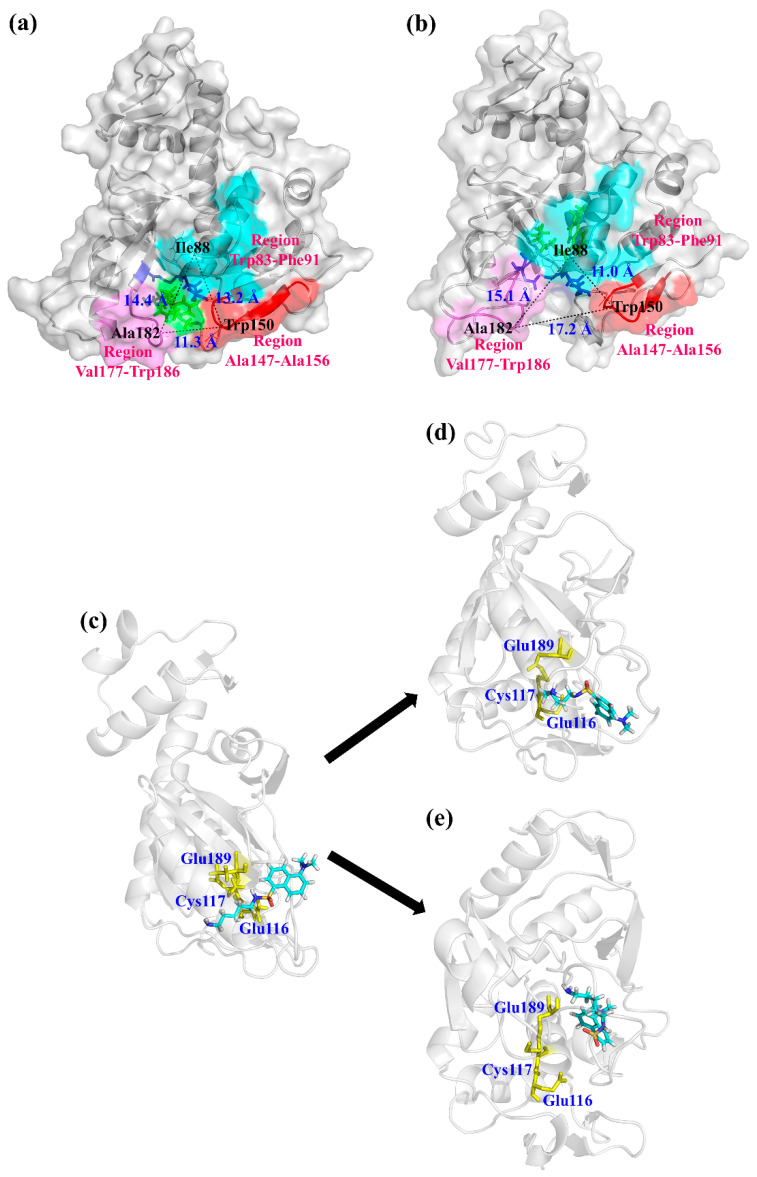
The structural illustration of BCETG-MDC at (**a**) 323 K and (**b**) 353 K. (**a**) The front view for the conformation of BCETG-MDC at 323 K at the end of molecular dynamics simulation. (**b**) The front view for the conformation of BCETG-MDC at 353 K at the end of molecular dynamics simulation. The Trp83-Phe91, Ala147-Ala156, and Val177-Trp186 regions are labeled in cyan, red, and magenta, respectively. The structure of BCETG is exhibited in gray. (**c**) The side view for the conformation of BCETG-MDC at the beginning of molecular dynamics simulation. (**d**) The side view for the conformation of BCETG-MDC at 323 K at the end of molecular dynamics simulation. (**e**) The side view for the conformation of BCETG-MDC at 353 K at the end of molecular dynamics simulation. The active center is colored in yellow. The structure of BCETG and MDC are shown in gray and cyan, respectively.

During MD simulation, the root-mean-square fluctuation (RMSF) values of every amino acid residue at 50 °C and 80 °C were analyzed and represented to determine the sensitive part of BCETG with the increasing temperature (Figure 4b). Comparing to BCETG at 50 °C, the two regions of Trp83-Phe91 and Ala147-Ala156 exhibited an observable increase in RMSF values at 80 °C, suggesting that the two regions demonstrated enhanced flexibility from 50 °C to 80 °C. The Ala147-Ala156 region, which was adjacent to the Trp83-Phe91 region, was situated at one side of the substrate channeling tunnel of BCETG (Figure 5a,b). Leu88 and Trp150, which were both in the loop region, were the two typical residues demonstrating the largest enhancement of RMSF values in each region. As exhibited in Figure 5a,b, the two regions of Trp83-Phe91 and Ala147-Ala156 were closer to each other at 80 °C than 50 °C. However, the two regions were far from another region, Val177-Trp186 (the other side of the tunnel), resulting in the expansion of the tunnel entrance of the substrate MDC and the change in interaction between the active sites of BCETG and substrate MDC (Figure 5a,b). As suggested in Figure 5c, a short distance was discovered between the active center of BCTG and substrate MDC at the beginning of the MD simulation. Substrate MDC showed similar positions at 50 °C (Figure 5d) in the end. By contrast, substrate MDC was far from the active center of BCETG at 80 °C and moved into the inside of BCETG in the end (Figure 5e). In addition, it was clear that the distance between MDC and BCETG’s active center was enhanced with the temperature raising from 50 °C to 80 °C during 15–100 ns of MD simulation (Figure S3). At 80 °C, the distance was quickly enhanced to approximately 0.29 Å from an initial 0.25 Å, and subsequently fluctuated close to 0.36 Å, suggesting the removal of the substrate from the active center. Hence, the WT activity might be remarkably destroyed at 80 °C. Nevertheless, the distance kept stable in the process of the whole simulation at 50 °C, demonstrating the stable manifold structure of the BCETG and MDC. Therefore, we speculated that the conformation change of critical regions Trp83-Phe91 and Ala147-Ala156 at the high temperature indeed enlarged the substrate tunnel entrance, leading to the substrate MDC being ‘beyond control’. This indicated that substrate MDC not only was far away from the active center of BCETG, but also entered into the BCETG’s structure interior, leading to the decrease in catalytic activity of BCETG and the impediment of substrate MDC release and enzyme regeneration. Thus, the two Trp83-Phe91 and Ala147-Ala156 regions of BCETG could be modified to improve its high-temperature activity in future research.

### 3.5. Electrophoresis Analysis of Protein Substrates Crosslinked by BCETG

**Figure 6:**
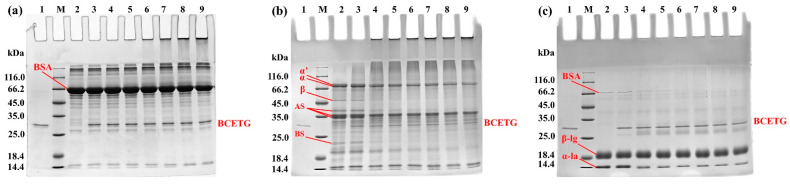
The electrophoretic arrangements of the native and BCETG-treated (**a**) BSA, (**b**) SPI, and (**c**) WP. Lane M, protein standard ladder; Lane 1, the purified BCETG; Lane 2, the native BSA, SPI, or WP; Lane 3–9, BCETG-treated BSA, SPI, or WP for 0–6 h.

SDS-PAGE was utilized to monitor the changes that occurred in the course of BSA, SPI, and WP treated with BCETG (Figure 6) for different times (1–6 h). A remarkable difference was noticed among the samples treated with various times. It was observed that a gradual loss of monomers simultaneously occurred with intensifying higher molecular-weight bands. However, some crosslinked proteins still stayed in the stacking gel and could not get into the resolving gel (Figure 6). As demonstrated in Figure 6a, the SDS-PAGE profiles of the BCETG-treated BSA samples were similar to that of MTG-treated samples [17,18,19]. The primary components of SPI (Figure 6b, lane 2) are 7S globulin, which is a trimer containing three subunits such as α, α′, and β subunits (45 kDa to 116 kDa), and 11S globulin chiefly composed of basic subunits and acidic subunits (18.4–45 kDa). The results demonstrate that BCETG could crosslink almost all components in SPI (Figure 6b, lane 4–9). However, no BCETG preference was observed in the crosslinking reaction for the protein ingredients of SPI. However, the BS in 11S globulin was hardly crosslinked by MTG [15]. The untreated WP showed three main bands (∼14 kDa, ∼18 kDa, and ∼67 kDa) on the SDS-PAGE gel, matching α-la, β-lg, and BSA monomers, respectively (Figure 6c, lane 2). WPs crosslinked by BCETG resulted in the slow reduction in α-la, β-lg, and BSA amounts with the increasing incubation time, suggesting that BCETG could effectively catalyze the three components in WP (Figure 6c, lane 4–9). Nevertheless, α-la was the major substrate in WPI for MTG, which showed a weak capability of crosslinking β-lg and BSA [17,18,20]. These results indicate BCETG showed differing substrate preferences from MTG, which could be owing to the various structures of proteins. Hence, it suggested that BCETG and other *Bacillus* TGs would be potential candidates for supplementing MTG functions in the food-processing industry. 

### 3.6. Crosslinked Amino Acid Preference in Protein Substrates by BCETG Treatment 

So far, the crosslinking mechanism of TGs at the protein level has not been investigated; thus, it is difficult to obtain the real abilities of TGs to promote the crosslinking of proteins with different characteristics, as well as rationally modulate and control the functional properties of protein matrices. Hence, the application of TG in food processing could be benefited by revealing the crosslinked sites in proteins. LC-MS/MS was utilized to determine the crosslinked sites in BSA, SPI, and WP by BCETG through identifying the crosslinked peptides. BCETG-treated BSA was taken as an example, and the characterization results of ADEKKFWGK and QNCDQFEK peptides are demonstrated in Figure S4. Figure S4a shows the extraction ion chromatogram of the crosslinked peptide ADEKKFWGK-QNCDQFEK and the uncrosslinked QNCDQFEK. However, uncrosslinked ADEKKFWGK was not observed. The mass spectra of the ADEKKFWGK-QNCDQFEK and QNCDQFEK peptides are presented in Figure S4b,c, respectively. The mass obtained from the experiment was consistent with the calculated theoretical monoisotopic mass.

**Figure 7:**
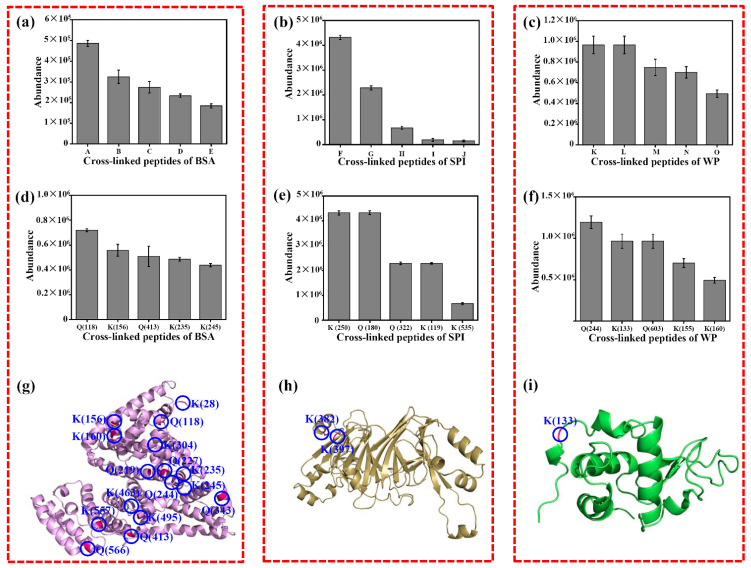
The crosslinking sites in the crosslinked BSA, SPI, and WP were analyzed by BCTG. (**a**) Five of the most plentiful peptides crosslinked in BSA were A (QEPER-FGERALKAWSVAR), B (ADEKKFWGK-QNCDQFEK), C (LSQKFPK-ATEEQLK), D (QEPER-ADEKKFWGK), and E (QNCDQFEK-TPVSEKVTK). (**b**) The five most plentiful peptides crosslinked in SPI were F (Glycinin G4/QQLQDSHQKIR-Seed linoleate 9S-lipoxygenase-2/KDQNSEK), G (Seed linoleate 13S-lipoxygenase-1/TDGQHILK-P34 probable thiol protease/DVSQQIKMANKK), H (Glycinin G4/AKSSSR-β-conglycinin alpha subunit 1/QSQVSELK), I (Glycinin G2/GRSQRPQDRHQK-Seed linoleate 9S-lipoxygenase/PLANGKGKVGK), and J (Glycinin G5/SQQQLQDSHQK-β-conglycinin alpha subunit 1/SSSRKTISSEDKPFNLR). (**c**) The five most plentiful peptides crosslinked in WP were: K (BSA/QEPER-ADEKKFWGK), L (α-lactalbumin/ALCSEKLDQWLCEK-BSA/LVVSTQTALA), M (β-lactoglobulin/TPEVDDEALEKFDK-BSA/NYQEAK), N (BSA/ADEKK-ALKAWSVARLSQK), and O (BSA/FWGKYLYEIARR-AWSVARLSQK). (**d**) The five most plentiful sites crossllinked in BSA: Q (118), K (156), Q (413), K (235), and K (245). (**e**) The five most plentiful sites crosslinked in SPI: Seed linoleate 13S-lipoxygenase-1/K (250), P24 oleosin isoform B/Q(180), Seed linoleate 13S-lipoxygenase-1/Q (322), P34 probable thiol protease/K (119), Glycinin G4/K (535). (**f**) The five most plentiful sites crosslinked in WP sample: BSA/K (244), α-lactalbumin/K (133), BSA/Q (603), BSA/K (155), and BSA/K (160). (**g**) The sites crosslinked in the tertiary structure of BSA containing K (28), Q (118), K (156), K (160), K (204), Q (219), Q (227), K (235), Q (244), K (245), K (303), Q (343), Q (413), K (463), K (495), K (557), and Q (566). (**h**) The sites crosslinked in the SPI-β-conglycinin alpha subunit 1 containing K (382) and K (397). (**i**) The sites crosslinked in the tertiary structure of WP-α-lactalbumin containing K (133).

Only a few Q and K sites were crosslinked in BSA, SPI, and WP. Figure 7 exhibits the investigation of crosslinked sites of BCETG-treated BSA, SPI, and WP. Figure 7a–c demonstrates the five most plentiful crosslinked peptides from BCETG-treated proteins. It was obvious that only a smaller number of them showed higher abundance than others, indicating that BCETG had remarkable site preference on crosslinking these proteins. Figure 7d–f presents the most plentiful crosslinked sites in the protein materials. As suggested in Figure 7d, the favorite cross-linking sites of BCETG were Q (118), K (156), Q (413), K (235) and K (245) on BSA. As exhibited in Figure 7e, the five favorite crosslinking sites of BCETG on soybean proteins were Seed linoleate 13S-lipoxygenase-1’s K (250), Seed linoleate 13S-lipoxygenase-1’s Q (322), P24 oleosin isoform B’s Q (180), Glycinin G4’s K (535), and P34 probable thiol protease’s K (119), which were from differing SPI protein ingredients. As demonstrated in Figure 7f, the four favorite crosslinking sites of BCETG on WP were found in BSA, such as K (155), K (244), Q (603), and K (160), and some were distinct from those from BSA. The possible reason should be that BSA could be crosslinked with other protein constituents in WP. Additionally, one preferred crosslinked site of BCETG on WP were K (133), which was from α-lactalbumin. Figure 7g–i suggested that these sites were mostly on the surface of BSA, SPI, and WP. Based on the above results, the internal crosslinking mode of BCETG at the protein level could help to estimate its crosslinking ability and regulate its applied manner.

## 4. Conclusions

In brief, four novel TGs demonstrating better thermostability and pH stability than MTG were exploited from *Bacillus* strains, which could offer different options for various food-processing processes. A structural analysis indicated that the Ala147-Ala156 region in the substrate tunnel of BCETG was related to its high temperature adaptability, which could be modified in further research. Furthermore, BCETG exhibited better preference for the protein constituents in SPI and WP than MTG, and the crosslinking mode of BCETG towards BSA, SPI, and WP displayed the intramolecular and intermolecular crosslinked path of glutamine and lysine in food protein substrates. Hence, novel protein crosslinking enzymes were provided in this study, which enhanced the understanding of the structure–activity relationship in new TGs associated with a high temperatures and further promoted its applications in food industry.

## Figures and Tables

**Table 1:** The summarized biochemical characterization of recombinant TGs.

Enzymes	Optimum Temperature	Optimum pH	Thermostability Range	pH Stability Range
BAMTG	50 °C	7.0	≤50 °C	5.0–9.0
BCETG	50 °C	6.0	≤50 °C	5.0–9.0
BSATG	50 °C	6.0	≤50 °C	5.0–9.0
BARTG	60 °C	6.0	≤50 °C	5.0–9.0

## Data Availability

The data used to support the findings of this study can be made available by the corresponding author upon request.

## Supplementary Materials

The following supporting information can be downloaded at https://www.mdpi.com/article/10.3390/foods11223722/s1. Table S1. Primers used in this study. Figure S1. Multiple alignments analysis of protein sequences for BAMTG, BCETG, BSATG, and BARTG with software DNAMAN. The similar amino acids were labeled in grey and the identical amino acids were highlighted in solid black. The active sites were presented in yellow color. Figure S2. The purified rTGs analyzed using SDS-PAGE. (a) Purified BAMTG. (b) Purified BCETG. (c) Purified BSATG. (d) Purified BARTG. Lane M: Standard protein ladder; Lane 1: purified BAMTG, BCETG, BSATG, and BARTG. Figure S3. Distance between the active center of BCETG and MDC as a function of time (100 ns) at 323 K and 353 K. Figure S4. LC-MS evaluation of the cross-linked peptides of ADEKKFWGK and QNCDQFEK from cross-linked BSA. (a) EIC of the cross-linked peptides ADEKKFWGK-QNCDQFEK and QNCDQFEK. (b) Mass spectrum of cross-linked peptide ADEKKFWGK-QNCDQFEK. (c) Mass spectrum of peptide QNCDQFEK.
