# Supplementary material for: Crosslinking Mechanism on a Novel Bacillus cereus Transglutaminase-Mediated Conjugation of Food Proteins
Source: Foods. 2022 Nov 19;11(22):3722. doi: 10.3390/foods11223722 (PMC9689123; doi:10.3390/foods11223722)
Supplement: Supplementary file 1 [file foods-11-03722-s001.zip › foods-1959177-supplementary.pdf]

Supplementary material

Crosslinking mechanism on a novel *Bacillus cereus* transglutaminase-mediated conjugation of food proteins

Hongbin Wang[1,†], Yuanfu Zhang[1,†], Zhaoting Yuan[1], Xiaotong Zou[1], Yuan Ji[1], Jiayi Hou[1], Jinfang Zhang[2,*],

Fuping Lu[1], Yihan Liu[1,*]

[1] Key Laboratory of Industrial Fermentation Microbiology, Ministry of Education, The College of Biotechnology, Tianjin University of Science and Technology, Tianjin 300457, China

[2] Tianjin Key Laboratory of Industrial Microbiology, Tianjin 300457, China

[†] Hongbin Wang and Yuanfu Zhang contributed equally to this work.

* Corresponding author:

E-mail address: lyh@tust.edu.cn (Y. H. Liu); jfzhang1992@sohu.com (J. F. Zhang).

Tel.: +86-022-6060-2949 (Y. H. Liu); +86-022-6060-1958 (J. F. Zhang)

Table S1. Primers used in this study.

Primers	Sequences
bamtg-F-NcoI	CATGCCATGGGCATTATCATATCCGGCCAAGT
bamtg-R-HindIII	CCCAAGCTTATGCATGATCTGATAAAGCG
bcetg-F-NcoI	CATGCCATGGGCATTGTAATAGGCCGTTCTATTGTAC
bcetg-R-NotI	AAGGAAAAAAGCGGCCGCATAAACTGTAGTTGTGTTGCCAAC
bsatg-F-NcoI	CATGCCATGGGCATTATCCTTTCAGGAAAGCC
bsatg-R-HindIII	CCCAAGCTTGCTGCCAAACATTTGCC
bartg-F-NcoI	CATGCCATGGGCATTAAAGTTAACCAACAAATTGTG
bartg-R-HindIII	CCCAAGCTTAGCTACATACGTAATCGACCC

Underline indicated restriction recognition sites.

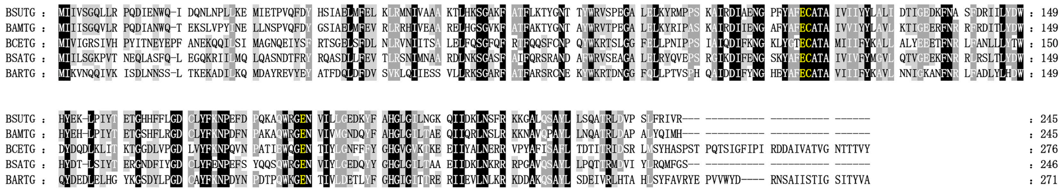

Figure S1. Multiple alignments analysis of protein sequences for BAMT, BCETG, BSATG, and BARTG with software DNAMAN. The similar amino acids were labeled in grey and the identical amino acids were highlighted in solid black. The active sites were presented in yellow color.

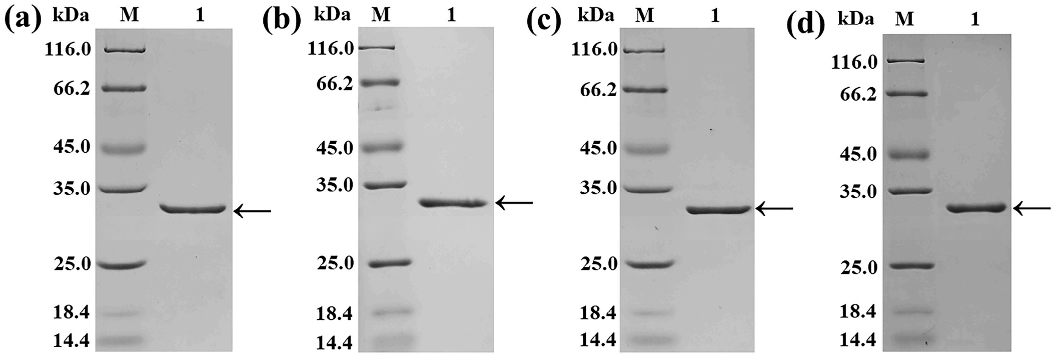

Figure S2. The purified rTGs analyzed using SDS-PAGE. (a) Purified BAMTG. (b) Purified BCETG. (c) Purified BSATG. (d) Purified BARTG. Lane M: Standard protein ladder; Lane 1: purified BAMTG, BCETG, BSATG, and BARTG.

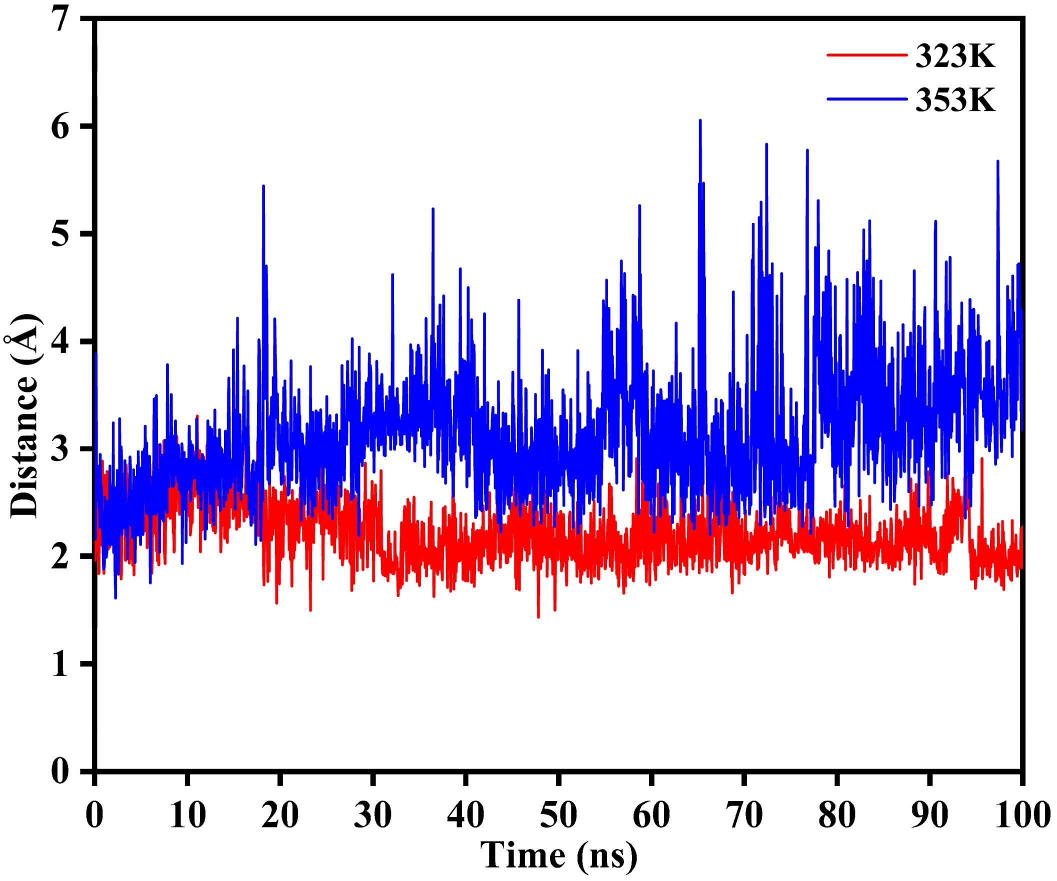

Figure S3. Distance between the active center of BCETG and MDC as a function of time (100 ns) at 323 K and 353 K.

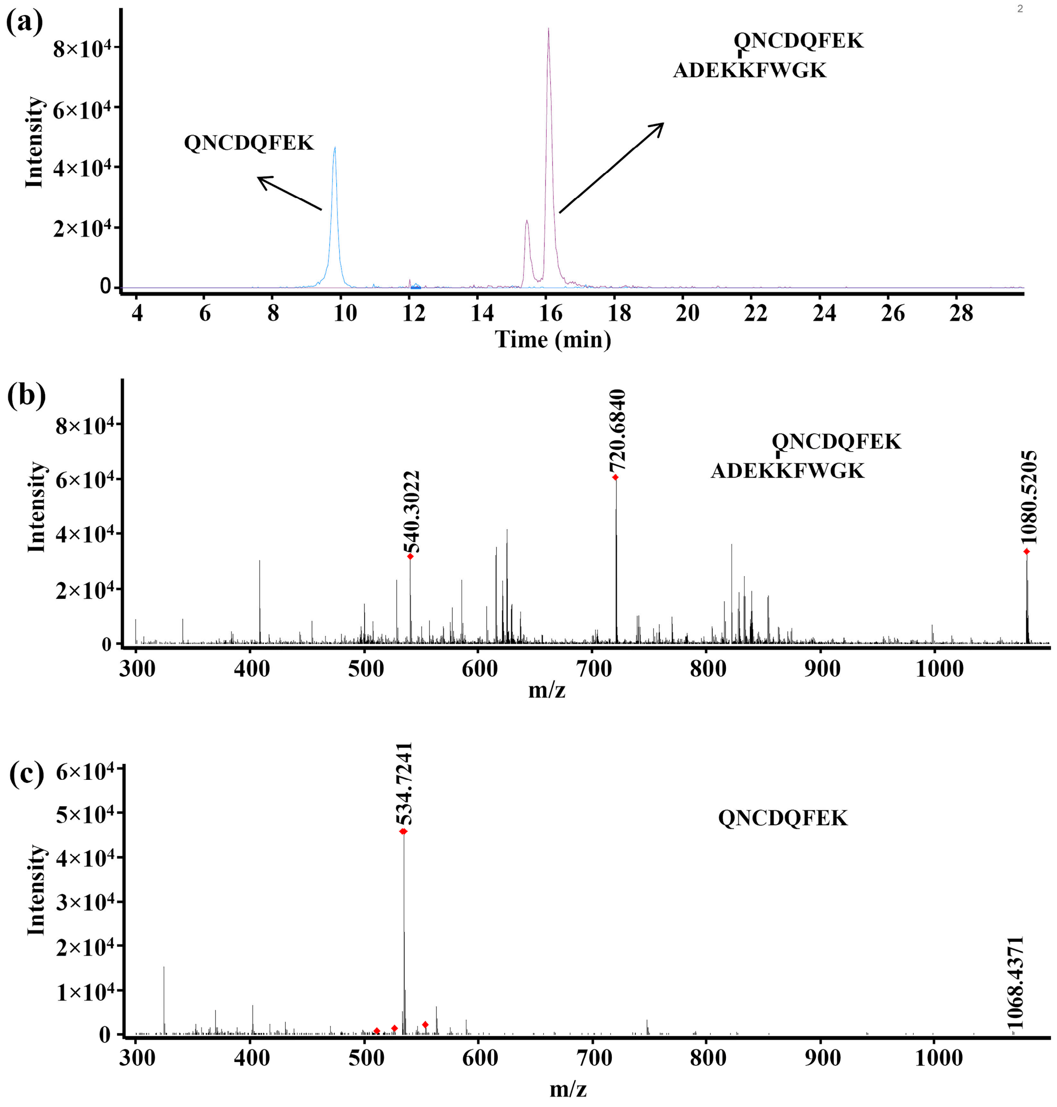

Figure S4. LC-MS evaluation of the cross-linked peptides of ADEKKFWGK and QNCDQFEK from cross-linked BSA. (a) EIC of the cross-linked peptides ADEKKFWGK-QNCDQFEK and QNCDQFEK. (b) Mass spectrum of cross-linked peptide ADEKKFWGK-QNCDQFEK. (c) Mass spectrum of peptide QNCDQFEK.
